# Reconstruction of perceived face images from brain activities based on multi-attribute constraints

**DOI:** 10.3389/fnins.2022.1015752

**Published:** 2022-10-26

**Authors:** Xiaoyuan Hou, Jing Zhao, Hui Zhang

**Affiliations:** ^1^School of Engineering Medicine, Beihang University, Beijing, China; ^2^School of Biological Science and Medical Engineering, Beihang University, Beijing, China; ^3^Key Laboratory of Biomechanics and Mechanobiology, Ministry of Education, Beihang University, Beijing, China; ^4^Key Laboratory of Big Data-Based Precision Medicine, Ministry of Industry and Information Technology of the People’s Republic of China, Beihang University, Beijing, China

**Keywords:** perceived face reconstruction, multi-conditional generative adversarial network, brain decoding, facial expression, functional MRI

## Abstract

Reconstruction of perceived faces from brain signals is a hot topic in brain decoding and an important application in the field of brain-computer interfaces. Existing methods do not fully consider the multiple facial attributes represented in face images, and their different activity patterns at multiple brain regions are often ignored, which causes the reconstruction performance very poor. In the current study, we propose an algorithmic framework that efficiently combines multiple face-selective brain regions for precise multi-attribute perceived face reconstruction. Our framework consists of three modules: a multi-task deep learning network (MTDLN), which is developed to simultaneously extract the multi-dimensional face features attributed to facial expression, identity and gender from one single face image, a set of linear regressions (LR), which is built to map the relationship between the multi-dimensional face features and the brain signals from multiple brain regions, and a multi-conditional generative adversarial network (mcGAN), which is used to generate the perceived face images constrained by the predicted multi-dimensional face features. We conduct extensive fMRI experiments to evaluate the reconstruction performance of our framework both subjectively and objectively. The results show that, compared with the traditional methods, our proposed framework better characterizes the multi-attribute face features in a face image, better predicts the face features from brain signals, and achieves better reconstruction performance of both seen and unseen face images in both visual effects and quantitative assessment. Moreover, besides the state-of-the-art intra-subject reconstruction performance, our proposed framework can also realize inter-subject face reconstruction to a certain extent.

## Introduction

Reconstruction of perceived objects from brain signals is a hot topic in brain decoding, and it is also an important application in the field of brain-computer interface these days. So far, researchers have been able to reconstruct perceived objects such as contours (Thirion et al., 2006; Miyawaki et al., 2008), colors (Brouwer and Heeger, 2009), numbers (Van Gerven et al., 2010), letters (Van Gerven et al., 2010; Schoenmakers et al., 2013; Du et al., 2017), natural scenes (Naselaris et al., 2009; Ren et al., 2021; Gaziv et al., 2022) and even dynamic movie clips (Nishimoto et al., 2011; Wen et al., 2018; Le et al., 2021). Among these reconstructed objects, the most exciting one is face. Face is the most important and complex “object” that we perceive in our daily life. Reconstruction of perceived faces has important practical significance in areas such as understanding the cognitive mechanisms, detecting the cognitive impairment and reconstructing suspects’ face in criminal investigation.

However, compared to common objects, reconstruction of perceived face faces many challenges. First, unlike common objects that contain mostly low-level visual features, the visual feature information of faces is focused on high-level, which is difficult to recover (Hershler and Hochstein, 2005). Second, unlike common objects that present only one kind of attribute, face can represent multiple facial attributes, such as expression, identity, gender, and so on (Gauthier et al., 2000). The reconstruction of faces should generate face images that is able to distinguish between these facial attributes. Third, unlike common objects elicit brain activities mainly in the primary visual cortex, faces elicit brain activities mostly in high level of visual cortex, which contain multiple local brain regions selective for different facial attributes (Haynes and Rees, 2006). The selection of these brain regions is key for perceived face reconstruction, but has never been addressed before.

In the early days, reconstructing perceived faces mainly used principal component analysis (PCA). In 2014, Cowen et al. used partial least squares (PLS) to setup relationship between face images and brain signals from human visual cortex. They are the first to realize the perceived face image reconstruction from the brain activities (Cowen et al., 2014). Lee and Kuhl (2016) used PCA to reconstruct perceived faces from visual and parietal cortex. In the same year, Nestor et al. (2016) combined PCA and multidimensional scaling (MDS) to reconstruct perceived face images. Chang and Tsao (2017) used PCA and linear regression to reconstruct face images from neural activities of monkey brain. With the rapid development of deep learning, using convolutional neural networks (CNN) and generative adversarial networks (GAN) as tools to reconstruct face images has been a very successful attempt (Güçlütürk et al., 2017; VanRullen and Reddy, 2019; Bao et al., 2020; Du et al., 2020; Higgins et al., 2021; Dado et al., 2022). Among these studies, the most representative work is VanRullen and Reddy (2019). The authors first used a variational auto-encoder (VAE) model to extract the face features from face image dataset, and then used linear regression to map the activities of the brain onto these face features. The face features were finally entered into a conditional GAN model for generating perceived faces. The method they used could clearly reconstruct perceived face image from the brain signals. However, there was still a gap between their reconstructed faces and the ground truth, especially in representing multiple facial attributes such as expressions and gender. To further improve the face reconstruction accuracy, a new algorithmic framework is needed for fully considering the multiple facial attributes and their associated neural activities at multiple face-selective brain regions.

In this study, we propose a framework for precise perceived face reconstruction. The framework consists of three modules: multi-task deep learning network (MTDLN), linear regression (LR) and multi-conditional generative adversarial network (mcGAN). The MTDLN is developed to simultaneously extract multi-dimensional face features attributed to facial expression, identity and gender from an integrated face image dataset. The LR maps brain signals from multiple face-selective brain regions to the multi-dimensional face features attributed to these multiple facial attributes. The mcGAN generates face image from the multi-dimensional face features predicted by the brain signals. The contribution of our proposed framework is threefold: First, the combination of information from facial identity, expression and gender, which characterize more detailed portrayal of human faces; Second, the selective use of brain signals from multiple brain regions of visual cortex, which encode multiple facial attribute representation; Third, the multiple constraints of GAN model based on multi-dimensional face features for more accurate face image reconstruction. We conducted extensive fMRI experiments to evaluate the reconstruction performance of our framework both subjectively and objectively. Our results showed that our proposed framework can achieve state-of-the-art reconstruction performance of both seen and unseen face images in both visual effects and quantitative assessment.

## Materials and methods

### The reconstruction framework

**FIGURE 1:**
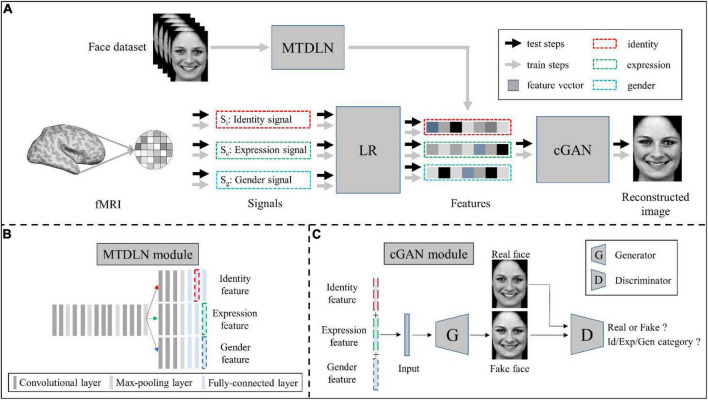
Schematic illustration of the perceived face reconstruction framework. **(A)** Algorithmic workflow. **(B)** The multi-task deep learning network (MTDLN) module. **(C)** The multi-conditional generative adversarial network (mcGAN) module.

Our face reconstruction framework consists of three modules: MTDLN, LR, and mcGAN (see Figure 1). MTDLN is the feature extraction module, which is developed purposely for simultaneously extracting the multi-dimensional face features attributed to expression, identity and gender. The LR set up relationship between the brain signals and the face features, which maps brain signals from multiple face-selective brain regions to the multi-dimensional face features. The mcGAN is the face generation module, which is used to reconstruct the perceived face images from the multi-dimensional face features predicted by the brain signals. Each module is developed in detail as follows.

#### Multi-task deep learning network

To extract the face features representing multiple attributes of facial expression, identity and gender without bias, we designed a multi-task network. The network has one input of a single face image, and three outputs to identify the face image’s expression, identity, and gender category, respectively (see Figure 1B). Previous studies have reported that the better the classification performance of the CNN model, the better the model’s ability to extract the object features (Tang et al., 2014; Sharma et al., 2018; Sahlol et al., 2020). In order to extract the more accurate multi-dimensional face features, we optimized both the multi-task CNN architecture and network parameters so that we can find a “best-performing” multi-task CNN model whose overall performance of classifying facial expression, identity and gender reach the best.

**FIGURE 2:**
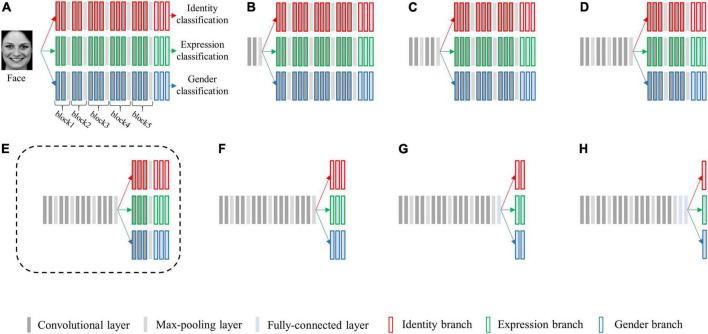
Schematic diagram of eight candidate multi-task network architectures for simultaneously classifying facial expression, identity, and gender. The architectures vary at which layer the network is split into three branches, sharing no layers **(A)**, one block **(B)**, two blocks **(C)**, three blocks **(D)**, four blocks **(E)**, five blocks **(F)**, five blocks and one fully-connected layer **(G)**, and five blocks and two fully connected layers **(H)**. Each bar represents one convolutional neural network (CNN) layer. Specifically, the bars with red borders represent layers in the identity branch, the bars with green borders represent layers in the expression branch, and the bars with blue borders represent layers in the gender branch. The architecture **(E)** surrounded by a dashed line is the best-performing architecture.

We selected the architecture of VGG-Face (Parkhi et al., 2015) as the basic single-task architecture and created a series of multi-task CNN architectures. The multi-task architectures differed in the layer at which the network is split into three branches. The five max-pooling layers (each separating one convolutional block) and the last three fully-connected layers were selected as the split layers, and a total of eight candidate multi-task network architectures were generated (see Figure 2). We replaced its first two fully-connected layers from 4,096 to 512 dimensions, and defined the last fully-connected layer of each output as 7, N and 2 dimensions, respectively. Here, seven represents the seven basic facial expressions: fear, anger, disgust, happiness, neutral, sadness, and surprise, N is the number of facial identities for re-training the model, two represents the gender categories of male and female.

For each candidate multi-task architecture, we fixed the parameters of model’s shared layers with pre-trained VGG-Face parameters, and re-trained the network parameters in layers of three separated branches with fine-tuning.

To characterize the relative weight between tasks during the multi-task learning, we defined a set of hyper-parameters (α1, α2, and α3) in multi-task CNN’s loss function as:


(1) $$L\,o\,s\,s=\alpha \,1^{*}\,l\,o\,s\,s\,1+\alpha \,2^{*}\,l\,o\,s\,s\,2+\alpha \,3^{*}\,l\,o\,s\,s\,3$$

where α1 + α2 + α3 = 1, lossi (*i* = 1, 2, 3) is the cross-entropy loss functions for facial expression, identity and gender classification tasks, respectively. To find the optimal set of hyper-parameters (α1, α2, and α3), we manually adjusted the settings of α1 from 0.1 to 0.9 at an interval of 0.1. Given gender is a more general representation of facial identity, we set α2 equals to α3. For each setting, we trained the parameters of the multi-task architecture and evaluated its overall classification performance across facial expression, identity, and gender.

Facial expression and gender classification accuracy were evaluated by comparing the predicted facial expression categories to their real labels across all face images in the validation set. Due to the “open-set test” (Scheirer et al., 2012), which means the facial identities used in the training and testing set are not allowed to be the same, facial identity classification accuracy was evaluated using the same strategy as FaceNet (Schroff et al., 2015). Specifically, we randomly selected 900 face image pairs belonging to the same identities and 900 face image pairs belonging to different identities from the validation set, then input the 1,800 face image pairs into the multi-task CNN models. We extracted the unit response patterns from the penultimate layer of multi-task CNN’s identity branch for each image pair and calculated the Euclidean distances. The 1,800 face image pairs were separated into 10 groups, nine used to define an Euclidean distance as the threshold and one used to judge if the pair of face images belonged to the same identity. We used 10-fold cross-validation to evaluate overall facial identity classification accuracy. The network parameters were set to train a fixed number of 500 epochs, and for each epoch, we calculated the classification accuracy for facial expression, identity and gender on validation set, respectively. After the training epochs reached a stable state, the peak classification accuracies of validation set were used to represent the performance of the optimized CNN model.

Of all the (α1, α2, and α3) settings, we found that when α1 = 0.4, α2 = α3 = 0.3, the multi-task network consistently achieved the peak classification accuracy (see Table 1). We therefore fixed this hyper-parameter setting for optimizing each of the multi-task architecture. By training each of the eight candidate multi-task CNN architectures defined above, we found that the multi-task model shared the first four blocks (the initial 11 convolutional layers) and separated the following layers achieved overall best classification performance (see Table 2). We termed this best-performing model as “MTDLN” for multi-dimensional face feature extraction in our reconstruction framework.

**TABLE 1:** Face classification performance of multi-task deep learning network (MTDLN) at varied task weights.

Weight settings			Accuracy		
Expression	Identity	Gender	Expression (%)	Identity (%)	Gender (%)
0.2	0.4	0.4	94.29	97.89	100
**0.4**	**0.3**	**0.3**	**95.71**	**98.00**	**100**
0.6	0.2	0.2	94.29	97.80	100
0.8	0.1	0.1	94.29	96.94	100

The bold text represents the best-performing task weight setting and the optimal classification performance.

**TABLE 2:** Face classification performance of multi-task deep learning network (MTDLN) at varied architectures.

Sharing blocks	Remaining blocks	Accuracy		
		Identity (%)	Expression (%)	Gender (%)
0	8	95.14	21.43	55
1	7	96.72	27.14	55
2	6	95.76	86.43	58.57
3	5	95.76	96.43	100
**4**	**4**	**98**	**95.71**	**100**
5	3	95.86	82.14	99.29
6	2	95.86	85.00	99.29
7	1	95.84	85.00	99.29

The bold text represents the best-performing architecture and the optimal classification performance.

The network parameters were trained with the stochastic gradient descent (SGD) optimization algorithm. The initial learning rate was set to 0.001, the batch size was set to 32. Each network was iterated for 500 epochs until it reached a stable state. To reduce the risk of over-fitting, we implemented the L2-regularization with the regularization rate of 0.001 for the first fully-connected layer (in each of the three branches), and set the dropout rate to be 0.5 for the second fully-connected layer (in each of the three branches).

#### Multi-conditional generative adversarial networks

Conditional generative adversarial networks (cGAN) (Mirza and Osindero, 2014) has been used for face image generation with conditional constrains (Chen et al., 2018; Lu et al., 2018; Bi et al., 2019; Deng et al., 2020; Heo et al., 2021). Compared to traditional GAN (Goodfellow et al., 2020), it allows use of additional information as latent variable input to constrain the image generation process. Here we designed a multi-conditional GAN (mcGAN) model, where face features representing multiple facial attributes were introduced as multiple constraint conditions. Therefore the mcGAN can realize more precise face image reconstruction with the desired facial attributes.

**FIGURE 3:**
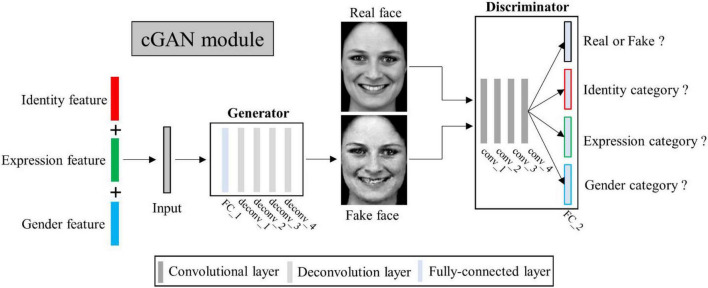
Illustration of multi-conditional generative adversarial network (mcGAN) module structure.


Our proposed mcGAN module was developed on the basis of deep convolutional generative adversarial networks (DCGANs) (Radford et al., 2015; see Figure 3). For the discriminator network, we added three fully-connected layers at the end of the fourth convolutional layers paralleling the original fully-connected layer, so that for each given face image, the discriminator network can justify (1) is the image real or fake? (2) Which expression category the face belongs to? (3) Which identity category the face belongs to? (4) What gender the face is? These improvements allow the discriminator to distinguish not only real or generated face images, but between categories of multiple face attributes. The loss function of the improved discriminator is defined as:

$$\underset{D}{m\,a\,x}L_{G\,A\,N}^{D}=E_{x∼p_{d}\,\left(x\right)}\;[l\,o\,g\,D\,\left(x\right)]+E_{z∼p_{z}\,\left(z\right)}\,\;[l\,o\,g\,\left(1-D\,\left(G\,\left(z\right)\right)\right)]$$

$$-\lambda_{D}\{L_{B\,C\,E}\left(t_{i\,d},D_{c\,l\,a\,s\,s_{i\,d}}\left(x\right)\right)+L_{B\,C\,E}\left(t_{i\,d},D_{c\,l\,a\,s\,s_{i\,d}}\left(G\left(z\right)\right)\right)$$

$$+L_{B\,C\,E}\,\left(t_{e\,x\,p},D_{c\,l\,a\,s\,s_{e\,x\,p}}\,\left(x\right)\right)+L_{B\,C\,E}\,\left(t_{e\,x\,p},D_{c\,l\,a\,s\,s_{e\,x\,p}}\,\left(G\,\left(z\right)\right)\right)$$

(2) $$+L_{B\,C\,E}\left(t_{g\,e\,n},D_{c\,l\,a\,s\,s_{g\,e\,n}}\left(x\right)\right)+L_{B\,C\,E}\left(t_{g\,e\,n},D_{c\,l\,a\,s\,s_{g\,e\,n}}\left(G\left(z\right)\right)\right)\}$$

where t_id_ is the feature vector attributed to facial identity extracted from MTDLN, t_exp_ is the feature vector attributed to facial expression, t_gen_ is the feature vector attributed to gender, and λ_D_ is weight parameter. *Z* is the concatenation of t_id_, t_exp_, and t_gen_, L_*BCE*_ (⋅) denotes the binary cross-entropy function. *x* represents the real image and G(z) is the corresponding generated one.

For the generator network, we added one fully-connected layer before the first deconvolution layer, so that the network can generate face images with the same size as input image from three latent feature vectors. Besides, we added a mean absolute error constraint, so that our generator can yield image more similar to the real image. The loss function of the improved generator is defined as:


(3) $$\underset{G}{m\,i\,n}L_{G\,A\,N}^{G}=E_{z∼p_{z}\,\left(z\right)}\,\;[l\,o\,g\,\left(1-D\,\left(G\,\left(z\right)\right)\right)]+\lambda_{G}\,L_{M\,A\,E}\,\left(G\,\left(z\right),x\right)$$

where λ_G_ is the weight parameter, *x* represents the real image and G(z) is the corresponding generated one, L_*MAE*_ (⋅) denotes the mean absolute error function. To select the most appropriate hyper-parameters of λ_*D*_ and λ_*G*_, we initially set the value of λ_*D*_ to 10, and then gradually increased the value to 50 at an interval of 10, so does for the selection of λ_*G*_. By comparing the model reconstruction performance at each setting, we found that the model performed best when λ_*D*_ and λ_*G*_ were both set to 20. We therefore set the fixed value of 20 for λ_D_ and λ_G_. To reduce the risk of over-fitting, we adopted the one-side label smoothing by setting the true label target value to 0.9, allowing the discriminator to learn more effectively to respond to generator attacks. In addition, we set the batch size to 16, which is relatively small and corresponds to introducing randomness that makes mcGAN harder to overfit.

To verify the effectiveness of using conditional GAN in generating face images with certain kinds of attributes, we first conducted an ablation experiment by randomly generating a series of white-noise data *N(0,1)*, and entered these white-noise data into the mcGAN for image reconstruction. The reconstructed images are shown in Supplementary Figure 2, and the quantitative evaluation of reconstruction performance are shown in Supplementary Table 9. It is clearly shown in the figure and table that using the randomly generated noise as latent variable input does not produce any desired results.

To further verify the improvements of proposed discriminator and generator, we conducted ablation experiments. We used the following four methods to generate the face images: (1) traditional cGAN model; (2) a cGAN model with proposed generator while keeping traditional discriminator; (3) a cGAN model with proposed discriminator while keeping traditional generator; (4) proposed mcGAN. To make a direct comparison, the input to these models used multi-dimensional face features that were extracted from MTDLN. The experimental results are shown in Table 3. We can see that the proposed mcGAN achieved the best performance among all four methods, indicating the effectiveness of mcGAN in precise face image generation.

**TABLE 3:** Face reconstruction performance with varied generator and discriminator of generative adversarial networks (GAN) model.

	MSE	SSIM	PSNR
Traditional cGAN	3,276.81 ± 1,279.14	0.31 ± 0.04	13.28 ± 1.6
cGAN with improved discriminator	3,370.04 ± 1,280.61	0.35 ± 0.06	13.61 ± 1.61
cGAN with improved generator	3,468.39 ± 877.81	0.36 ± 0.05	12.87 ± 1.09
mcGAN	**2,050.66 ± 760.42**	**0.53 ± 0.06**	**15.28 ± 1.53**

The bold text represents the optimal reconstruction performance.

#### Linear regression

We used a set of linear regression models to establish the mappings between the brain signals and the multi-dimensional face features. Previous neuroimaging studies have shown that face visual stimuli evoked several brain face-selective regions in visual cortex, and these regions showed varied preferences to facial expression, identity, and gender (Pessoa et al., 2002, 2006; Pitcher, 2014; Zhang et al., 2016; Shao et al., 2017). Based on these cognitive evidences, we created three groups of brain ROIs, each representing one facial attribute, we then established the linear relationship between brain signals of each ROI group and the face features of each certain face attribute, respectively. Specifically, the mapping between expression ROI group and its corresponding expression features is formulated as:


(4) $$T_{e\,x\,p}=S_{e\,x\,p}\;W_{exp}$$

the mapping between identity ROI group and its corresponding identity features is formulated as:


(5) $$T_{i\,d}=S_{i\,d}\;W_{i\,d}$$

and the mapping between gender ROI group and its corresponding gender features is formulated as:


(6) $$T_{g\,e\,n}=S_{g\,e\,n}\;W_{g\,e\,n}$$

In the above three formulas, *T* is the vector of face features. *S* is the brain signals from brain regions. *W* is the weight matrix that links the brain signals to the face features. *W* can be estimated with the following formula in the training phase:


(7) $$W={\left(S^{T}\,S\right)}^{-1}\,S^{T}\,T$$

#### Reconstruction workflow

For the three modules of our proposed framework, the MTDLN and mcGAN were trained by using only face image dataset and no brain signals were required, while training the LR required brain signals for the participation. Specifically, there were three steps to train the proposed framework. First, a multi-labeled face dataset was used to train the MTDLN, and the multi-dimensional face features were extracted from the MTDLN’s last two layers; Second, the multi-dimensional face features were used as conditional constraints to train the mcGAN; Third, the brain signals from multiple brain ROIs and the multi-dimensional face features were used to train the LR module. After all the parameters of the framework were well-trained, face images were reconstructed by inputting the testing brain signals into the framework. The prediction steps were as follows: an independent set of brain signals were entered into the LR module, and the multi-dimensional face features were predicted. These features as constraints were then entered into mcGAN to achieve precise face image reconstruction.

### Multi-label face image dataset

Our face stimulus dataset consisted of 952 front view face images that belong to 136 facial identities, each depicting seven facial expressions: fear, anger, disgust, happiness, neutral, sadness, and surprise, 60 individuals are female. These face images were originally from Karolinska Directed Emotional Faces (KDEF) dataset (Lundqvist et al., 1998) and Radboud Faces Database (RaFD) dataset (Langner et al., 2010), and were converted to gray-scale, normalized to have equivalent size, luminance and contrast, and resized to 330 × 450 pixels, to minimize the low-level visual differences. In total, 63 images were removed without further analysis because these face images represent children where gender could not be easily identified. The following images were divided into training and validation dataset at a rough ratio of 5:1, where the training dataset containing 749 images was used to optimize the MTDLN and mcGAN, and the validation dataset containing 140 images was further divided into two parts, one for optimizing the LR and the other for predicting face images.

### Functional magnetic resonance imaging data collection

Two healthy subjects (S1: male, 22-year-old; S2: female, 28-year-old) participated in an event-related fMRI experiment, in which each was shown 952 face images five times in five sessions of 68 runs, taking a total of 12.5 scan hours for each subject. During scanning, subjects performed one-back matching tasks (pressing the left button if the current face image matched the preceding one, and the right button if it did not) while they viewed the face images. Each trial began with one of the 952 face images for 1 s, followed by a gray cross fixation centered on the black screen for a random duration of 4–6 s. The order of the face images was randomized across the whole scans.

Besides the main fMRI experiment scans, each subject also performed an independent face localizer fMRI experiment to identify each individual’s face-selective regions. During the face localizer runs, subjects viewed blocks of human faces, common objects and scrambled images, and were asked to press the left button if the current image matched the preceding one, and the right button if it did not (one-back matching task). Each block lasted 24 s, with a 16-s blank period between blocks. Within a block, each image was presented for 500 ms, with 1-s blank period between images. There were two blocks for each condition per run, and the order of the blocks was randomized. Each localizer run lasted 5 mins. The face images used in the localizer runs were all neutral faces, and they differed from those used in the main experiment. The face-selective regions were defined in each individual subject by contrasting the fMRI response to faces with that to objects (*p* < 0.001, uncorrected). Our identified face-selective regions included occipital face area (OFA), fusiform face area (FFA), anterior inferotemporal cortex (aIT), posterior superior temporal sulcus (pSTS) and amygdala, which was quite consistent with many previous studies localized face-selective regions (Kanwisher et al., 1997; McCarthy et al., 1997; Harris et al., 2012, 2014). The brain regions in primary visual cortex (V1) were also included. These ROIs were defined by drawing a sphere with a radius of 6 mm (roughly including 56 voxels) around the activation peak or within the primary visual cortex. The schematic diagram and the location for each ROI are shown in the Supplementary Figure 1 and Supplementary Table 10. All participants gave informed consent according to a protocol approved by the research ethics committee at institute of biophysics, Chinese Academy of Sciences.

Imaging data were collected using a Siemens 3.0 Tesla scanner with an 8-channel head coil. The functional images were acquired with a single-shot interleaved gradient-recalled echo planar imaging sequence to cover the whole brain (TE = 30 ms; TR = 1,500 ms; flip angle = 70°; matrix size = 72 × 72; voxel size = 2 mm^3^ × 2 mm^3^ × 2 mm^3^; 72 oblique axial slices). High-resolution anatomical images were also acquired from each subject (1 mm × 1 mm × 1 mm voxels, TR = 2,350 ms, TE = 3.14 ms, 176 sagittal slices).

fMRI data were preprocessed as follows: Data from the first four TRs from each run were discarded. The remaining volumes were de-obliqued, slice-time corrected, realigned, normalized to the mean signal value. A gamma function with a peak of one was used for the hemodynamic response function, and a general linear model was established for each of the 952 face images for each scan run. The baseline and head movement parameters were regressed out in the general linear model (GLM). The parameter estimates of the hemodynamic response evoked by each face at each voxel were extracted in the identified face-selective ROIs.

### Reconstruction performance evaluation

To quantify the performance of the perceived face reconstruction, we used several methods to evaluate the reconstruction performance objectively and subjectively.

#### Objective evaluation

For objective evaluation, we used a series of evaluation matrix: mean square error (MSE), peak signal-to-noise ratio (PSNR), and structural similarity (SSIM) metrics, to evaluate the reconstruction performance (Wang et al., 2004). Specifically, MSE measures the distance between the reconstructed image and the ground truth in pixel space, which can be calculated as:


(8) $$M\,S\,E=\frac{1}{N}\,\sum_{i}^{N}{\left(x_{i}-y_{i}\right)}^{2}$$

where *x* is the reconstructed image, *y* is the ground truth image, and n is the number of image pixels. PSNR measures the ratio between the maximum possible value and the power of distorting noise value that affects the quality of the image, which can be calculated as:


(9) $$P\,S\,N\,R=10\,l\,o\,g_{10}\,\frac{M\,A\,X^{2}}{M\,S\,E}$$

where MAX is 255 in our experiment. PSNR can be considered as a deformation of MSE, and the higher its value, the better the quality of the reconstructed image. SSIM is used to quantify the perceived image quality where image structure is taken more into account. SSIM is calculated as:


(10) $$\text{SSIM}=\frac{\left(2\,\mu_{x}\,\mu_{y}+C_{1}\right)\,\left(2\,\sigma_{x\,y}+C_{2}\right)}{\left(\mu_{x}^{2}+\mu_{y}^{2}+C_{1}\right)\,\left(\sigma_{x}^{2}+\sigma_{y}^{2}+C_{2}\right)}$$

where μ_*x*_ and μ_*y*_ denote the mean intensity values of x and y, respectively; σ_*x*_^2^ and σ_*y*_^2^ denote the variances of x and y, σ_*xy*_ denotes the covariance of x and y, C_1_ and C_2_ are constants. The SSIM value, scale 0–1 and is one only if the reconstructed image is identical to the ground truth.

To statistically test the evaluation matrix, e.g., SSIM, we defined a “SSIM accuracy”. For each reconstructed face image, we first calculated the SSIM value between the ground truth (original face image stimulus) and the reconstructed image as “SSIM1”. We then randomly picked a face image in the dataset other than the ground truth (distractor), and calculated the SSIM value between the distractor image and the reconstructed image as “SSIM2”. Reconstruction was successful and was assigned to 1 when SSIM1 was higher than SSIM2, and reconstruction failed and was assigned to 0 when SSIM1 was lower than SSIM2. The “SSIM accuracy” was calculated as the proportion of successful reconstruction pairs among all 140 testing image pairs. We repeated this procedure 40 times, and obtained 40 “SSIM accuracy” observers for each reconstructed face image. We then summarized the SSIM accuracy across all reconstructed face images to perform a one-sample *t*-test against the null hypothesis of 0.5 SSIM accuracy value (FDR corrected).

#### Subjective evaluation

To evaluate the reconstruction performance more comprehensively, we also conducted the subjective evaluation. We recruited 30 participants to assess the similarity between the reconstructed face images and their ground truth. Participants were presented with samples of paired images (reconstructed face image and its ground truth), and were instructed to rate the degree to which the reconstructed faces look similar to the original images by giving empirical scores from 0 to 10, in the perspectives of identity, expression and gender. 10 means that the participant is confident that the face image pair is from the same face category, 5 means that the participant is uncertain if the face image pair belongs the same face category or not, and 0 means the participant is confident that the face image pair is from totally two different face categories. For each reconstructed face image, we got 30 empirical scores for each attribute. These empirical scores were statically evaluated by performing a one-sample *t*-test against the null hypothesis of an empirical score of 5. The same procedure was used to subjectively test the reconstruction performance of facial expression, identity and gender, respectively.

## Experiments and results

### Reconstruction from face features

To estimate the effectiveness of our proposed MTDLN module in extracting the multi-dimensional face features, and its impact on the reconstruction performance, we first reconstructed face images with the multi-dimensional face features that were extracted directly from MTDLN, which means this part of the experiment did not involve any brain signal.

Besides MTDLN, we also examined four commonly used feature extraction models that appeared in previous references (Parkhi et al., 2015; Chang and Tsao, 2017; Ming et al., 2019; VanRullen and Reddy, 2019), and compared these models’ performance with that of MTDLN in reconstructing perceived face images. These models included PCA (principle component analysis), VAE (variational autoencoder), pre-trained VGG-Face, and re-trained VGG-Face. For PCA (Chang and Tsao, 2017), we used the face images in the training dataset to span a 749 dimensional eigen-face space, and calculated the “eigen-score” vector of the first 521 (consistent with MTDLN feature dimension) principal components for each face image as the face feature. The eigen-score face feature was then used as a conditional constrain on the cCAN model for face image generation. For VAE, we used a pre-trained variational autoencoder described in VanRullen and Reddy (2019) to extract the 1,024-dimensional latent vector for each face image. The latent vector was then further used as conditional constrain to be entered into cCAN for face image generation. For pre-trained VGG-Face, we extracted the 2622-dimensional feature vector from the last fully-connected layer of the pre-trained VGG-Face (Sahlol et al., 2020) to the cGAN model for face image reconstruction. For re-trained VGG-Face, we fine-tuned three single-task VGG-Face networks to perform facial expression, identity and gender classification tasks, respectively. For fair comparison with MTDLN, each single-task network’s initial 11 convolutional layers were fixed and only the late layers including one convolutional block and three fully-connected layers were re-trained. We then extracted the feature vectors from the last fully-connected layer of each of the three networks. These feature vectors were concatenated and were entered into cGAN model for face image reconstruction.

**FIGURE 4:**
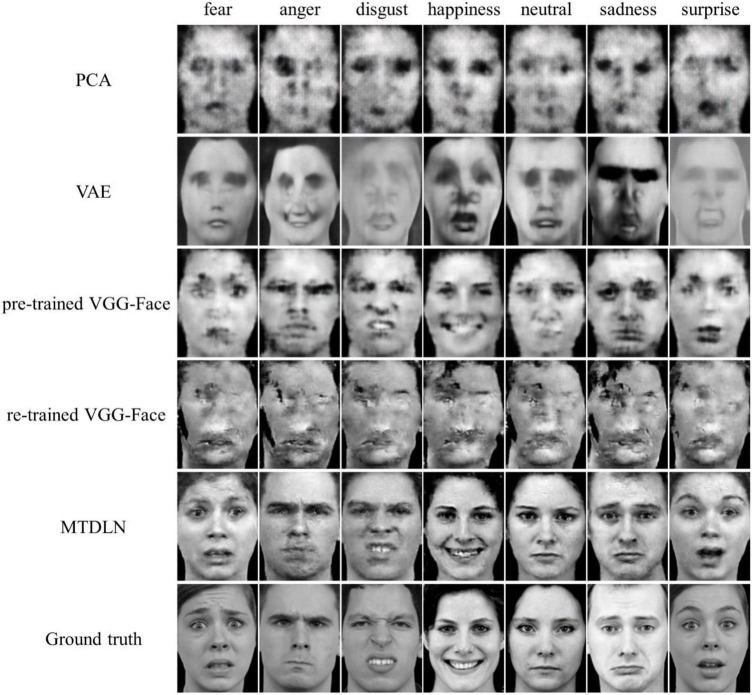
Representative samples of reconstructed faces from five different feature-extraction models. From top row to bottom row: PCA, VAE (VanRullen and Reddy, 2019), pre-trained VGG-Face, re-trained VGG-Face, multi-task deep learning network (MTDLN), and Ground truth.

The reconstructed faces and their evaluation metrics are illustrated in Figure 4 and Table 4. As shown in the figure, the face images reconstructed by PCA contained only face contours and their facial attributes of expression, identity, and gender were unrecognizable. The face images reconstructed by VAE and pre-trained VGG-Face contained very blurry faces with facial attributes barely recognizable. Surprisingly, the re-trained VGG-Face failed to generate face images. This may due to the three-independent single-task VGG-Face networks that were not well-trained with our relatively small size of training dataset. By comparison, the proposed MTDLN were trained with the same size of training dataset, and the face images reconstructed by MTDLN represented multiple vivid facial attributes and were the closest to the ground truth among all extraction models. The quantitative results of evaluation metrics (see SSIM evaluation in Table 4 and all three evaluation metrics results of SSIM, MSE, and PSNR in Supplementary Table 1) show that re-trained VGG-Face performed worst and its reconstruction was significantly below chance level (*p* = 0.14). The reconstruction of PCA, VAE, pretrained VGG-Face and MTDLN were significantly above chance level (for all four models, *p* < 0.001, FDR corrected), in which MTDLN performed best among all the examined models. By subjective evaluation of the reconstruction performance with empirical scores from 30 recruited participants, we found that the faces generated by PCA and re-trained VGG-Face were inconsistent with ground truth in terms of all three facial attributes (*p* > 0.99). The faces generated by VAE and pre-trained VGG-face were consistent with ground truth in terms of gender, but inconsistent with ground truth in terms of expression and identity (VAE: expression *p* = 0.55; identity *p* = 0.90; gender *p* = 0.027; pre-trained VGG-Face: expression *p* < 0.001; identity *p* = 0.079; gender *p* < 0.001, FDR corrected). In contrast, the faces generated by MTDLN were clearly consistent with ground truth in terms of all three attributes of expression (*p* < 0.001), identity (*p* < 0.001) and gender (*p* < 0.001), *p* values were FDR corrected.

**TABLE 4:** Quantitative evaluation of reconstruction performance by using five different face-feature extraction models.

Methods	Objective evaluation	Subjective evaluation
	
	SSIM	Empirical scores (exp, id, and gen)
PCA	0.37 ± 0.05*	(2.69, 2.65, 3.44)
VAE	0.51 ± 0.08*	(4.95, 4.40, 6.00*)
pre-trained VGG-Face	0.45 ± 0.06*	(6.25*, 5.58, 7.46*)
re-trained VGG-Face	0.21 ± 0.06	(1.22, 1.01, 1.31)
MTDLN	0.53 ± 0.06*	(8.50*, 8.10*, 9.17*)

exp, expression; id, identity; gen: gender.

*p < 0.05.

### Reconstruction from brain activities

Our proposed framework can realize perceived face reconstruction not only within (intra-subject reconstruction) but also across subjects (inter-subject reconstruction). In the intra-subject reconstruction, we trained the reconstruction framework with brain signals from one subject, and predict the perceived faces with independent brain signals from the same subject. In the inter-subject reconstruction, we train the reconstruction framework from one subject’s brain signals and predict the perceived faces with another subject’s brain signals.

#### Intra-subject experiments

We used a set of fMRI signals from each individual subject to train its relationship with the multi-dimensional face features, and used an independent set of fMRI signals from the same subject to predict the face features. The predicted face features were then entered into the well-trained mcGAN for perceived face image reconstruction.

##### Reconstruction of seen images

**FIGURE 5:**
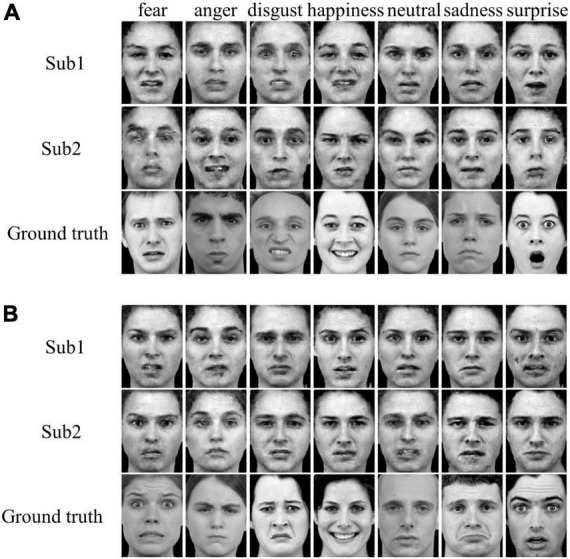
Intra-subject perceived face reconstruction from functional magnetic resonance imaging (fMRI) signals. **(A)** Reconstruction of seen faces from brain activities in each individual subject. **(B)** Reconstruction of unseen faces from brain activities in each individual subject. Sub1: subject 1 **(top row)**; Sub2: subject 2 **(middle row)**; Ground truth: original face image stimuli **(bottom row)**.

We used the brain neural responses evoked by the 140 face stimuli from three fMRI runs’ data to train the parameters of LR module, and used the neural responses evoked by the same 140 face stimuli from two remaining fMRI runs’ data to predict the multi-dimensional face features. Previous neuroimaging studies have reported that amygdala and pSTS located in the dorsal visual pathway were involved in coding facial expression, FFA and aIT in the ventral visual pathway were involved in coding facial identity (Pessoa et al., 2002, 2006; Pitcher, 2014; Zhang et al., 2016; Shao et al., 2017), OFA was involved in coding the holistic face information (Pitcher et al., 2007; Rhodes et al., 2009; Zhang et al., 2012; Shao et al., 2017), while V1 was involved in processing low-level visual information (Hubel and Wiesel, 1977; Magnussen, 2000; Benoit et al., 2010). Based on these cognitive evidences, we used a strategy to combine the ROIs according to their preference to certain facial attributes. ROIs were assigned into three groups, group 1 included V1, OFA, amygdala and pSTS, for decoding facial expression; group 2 included V1, OFA, FFA and aIT, for decoding facial identity; group 3 included ROIs that were the same as in group 2, for decoding gender. We trained linear regression parameters between brain signals from each ROI group and each dimension of face features representing one of the three face attributes, respectively. The predicted face features were concatenated to constitute the multi-dimensional face features and were further entered into the mcGAN for face image generation. Figure 5A shows the reconstructed face images from each of the two subjects’ fMRI signals, which appear realistic and quite similar to the ground truth from the visual point of view. The quantitative evaluation of the reconstruction performance with SSIM (see SSIM evaluation in Table 5A and all three evaluation metrics results of SSIM, MSE, and PSNR in Supplementary Table 3) indicates that our proposed framework can effectively reconstruct the perceived face images from individual subjects (*p* < 0.001 for both subject1 and subject2, FDR corrected). However, our subjective evaluation of the reconstruction with empirical scores shows that only gender can be significantly reconstructed, but not expression and identity (subject1: expression *p* = 0.15, identity *p* > 0.99, gender *p* < 0.001; subject2: expression *p* > 0.99, identity *p* > 0.99, gender *p* < 0.001, FDR corrected). In addition, we also reconstructed the seen face images from intra-subject fMRI signals using PCA, VAE, pre-trained VGG-Face, and re-trained VGG-Face. The quantitative evaluation of the reconstruction performance is shown in Supplementary Table 7. The representative samples of reconstructed seen faces are shown in Supplementary Figures 3, 4. According to the results, it is clearly shown that our proposed framework can achieve state-of-the-art reconstruction performance in both visual effects and quantitative assessment.

**TABLE 5:** Quantitative evaluation of seen (A) and unseen (B) face images reconstruction from intra-subject functional magnetic resonance imaging (fMRI) signals.

		Objective evaluation	Subjective evaluation
	
		SSIM	Empirical scores (exp, id, and gen)
A	Sub1	0.39 ± 0.08*	(5.27, 3.74, 6.10*)
	Sub2	0.38 ± 0.07*	(3.89, 4.05, 6.43*)
B	Sub1	0.40 ± 0.06*	(4.83, 2.96, 4.38)
	Sub2	0.38 ± 0.07*	(3.96, 4.00, 6.38*)

exp, expression; id, identity; gen, gender.

*p < 0.05.

To what extent such ROI combination strategy contribute to the final reconstruction performance? To verify this, we also tested two other strategies to combine ROIs. In Strategy 1, we extracted fMRI signals only from the primary visual cortex (V1). In Strategy 2, we extracted fMRI signals from all ROIs. We extracted the fMRI signals from these ROI combinations to predict the face features for face reconstruction. The reconstruction results for the three strategies of ROI combinations are shown in Table 6 (all three evaluation metrics results of SSIM, MSE, and PSNR in Supplementary Table 2). It can be observed that our proposed ROI combinations achieved better performance than the other two ROI combination strategies (paired *t*-test, proposed vs. Strategy1: *p* < 0.001 for subject1, *p* = 0.34 for subject2; proposed vs. Strategy 2: *p* = 0.089 for subject1, *p* = 0.0024 for subject2), indicating that the selective use of brain signals from attribute-sensitive brain regions improved the face reconstruction.

**TABLE 6:** Quantitative evaluation of reconstruction performance with three different ROI combination strategies.

	Brain ROIs	Subjects	SSIM
Strategy 1	id/gen/exp: V1	Sub1	0.36 ± 0.08
		Sub2	0.36 ± 0.07
Strategy 2	id/gen/exp: V1, OFA, amygdala, STS, FFA, and aIT	Sub1	0.38 ± 0.08
		Sub2	0.37 ± 0.07
Our proposed strategy	id/gen: V1, OFA, FFA, and aIT exp: V1, OFA, amygdala, and STS	Sub1	0.39 ± 0.08*
		Sub2	0.38 ± 0.07*

exp, expression; id, identity; gen, gender.

*p < 0.05.

Furthermore, we added a series of experiments to demonstrate how the face reconstruction performance could be improved by adding the gender or expression attributes. Firstly, we reconstructed the face images from all the brain regions relevant to face processing by using the framework with the identity attribute as the constraint (termed “Strategy 3”). Secondly, we reconstructed the face images from all the brain regions relevant to face processing by using the framework with identity and gender attributes as the constraints (termed “Strategy 4”). Thirdly, we reconstructed the face images from all the brain regions relevant to face processing by using the framework with identity and expression attributes as the constraints (termed “Strategy 5”). Fourthly, we reconstructed the face images from all the brain regions relevant to face processing by the framework with identity, expression and gender attributes as the constraints, which had been termed as “Strategy 2” in our initial manuscript. Together with the reconstruction of our selective use of brain signals from attribute-sensitive brain regions, we used the five different strategies as constraints to examine the reconstruction performance from brain signals. Finally, we compared the reconstruction performance among these five strategies.

Our results showed that, compared with Strategy 3, both Strategy 4 and Strategy 5 performed significantly better (*p* < 0.005, paired *t*-test), indicating that the constraints of expression and gender significantly contributed to the improvement of the reconstruction performance. Compared with Strategy 4, Strategy 2 performed significantly better (*p* < 0.005), indicating that the constraint of expression still significantly contributed to the improvement of the reconstruction performance with the constraints of identity and gender. Strategy 2 did not significantly outperform Strategy 5 (*p* = 0.793 for subject1, *p* = 0.757 for subject2), indicating that gender constraint did not significantly contribute to the improvement of reconstruction performance under the premise of identity and expression constraints. Compared with strategy 2, our proposed strategy performed significantly better (*p* < 0.005), indicating that the selective use of brain signals from attribute-sensitive brain regions significantly contribute to the improvement of reconstruction performance.

##### Reconstruction of unseen images

To further assess the robustness of our reconstruction framework, we challenged to reconstruct the unseen face images, which means the face images used to optimize LR parameters were not used for prediction. Of the 140 face stimuli, 126 face images and their corresponding fMRI signals from all 5 runs’ data were used to train the LR weight and the remaining 14 images’ fMRI data were used for reconstruction (10-fold cross validation). The fMRI signals were extracted from the three ROI groups as mentioned in the above sub-section “reconstruction of seen images”. Figure 5B shows the reconstructed face images, which are visually similar to the ground truth. For the quantitative assessments of the reconstruction, we compared the evaluation metrics of reconstructed face images with the ground truth, and found significant similarities between them (one-sample *t*-test, *p* < 0.001 for both subject1 and subject2, FDR corrected), indicating that the framework can also effectively reconstruct unseen face images (see SSIM evaluation in Table 5B and all three evaluation metrics results of SSIM, MSE, and PSNR in Supplementary Table 4). According to the empirical scores, we found gender but not expression and identity can be significantly recognized from the reconstructed images (one-sample *t*-test, subject1: expression *p* = 0.70, identity *p* > 0.99, gender *p* > 0.99; subject2: expression *p* > 0.99, identity *p* > 0.99, gender *p* < 0.001, FDR corrected). Overall, these results indicated that the proposed framework is highly robust and can reconstruct unseen face images with discriminative facial attributes from fMRI signals of multiple brain regions. In addition, we also reconstructed the unseen face images from intra-subject fMRI signals using PCA, VAE, pre-trained VGG-Face, and re-trained VGG-Face. The quantitative evaluation of the reconstruction performance is shown in Supplementary Table 8. The representative samples of reconstructed unseen faces are shown in Supplementary Figures 5, 6. According to the results, it is clearly shown that our proposed framework can achieve state-of-the-art reconstruction performance in both visual effects and quantitative assessment.

#### Inter-subject experiments

To explore the feasibility of establishing a reconstruction framework that can be generalized to different participants, we also conducted the inter-subject reconstruction experiment. We used one subject’s fMRI signals to train the reconstruction framework and another subject’s fMRI signals to reconstruct the perceived faces. Specifically, we used PCA to span a 56-dimensional eigen-space using the fMRI signals of one subject. We then used LR to establish the linear relationship between the eigen coordinates of the fMRI signals in this eigen-space and the corresponding image features, i.e., the fMRI signals of this subject are used to train our reconstruction framework. Similarly, a portion of the fMRI signals from another subject (test subject) was also used to span a 56-dimensional eigen-space using PCA, and then we built a transformation of the two eigen-spaces based on the neural responses elicited by the same visual stimuli in the same brain regions of both subjects. In this way, when using the test subject’s brain fMRI signals for prediction, we first transform the feature vector of the fMRI signals in its eigen-space to the eigen-space established by the framework training subject’s fMRI signals, and then predict the face image’s feature vector based on the feature coordinates of the signal in the eigen-space of the training framework, and achieve face reconstruction.

##### Reconstruction of seen images

**FIGURE 6:**
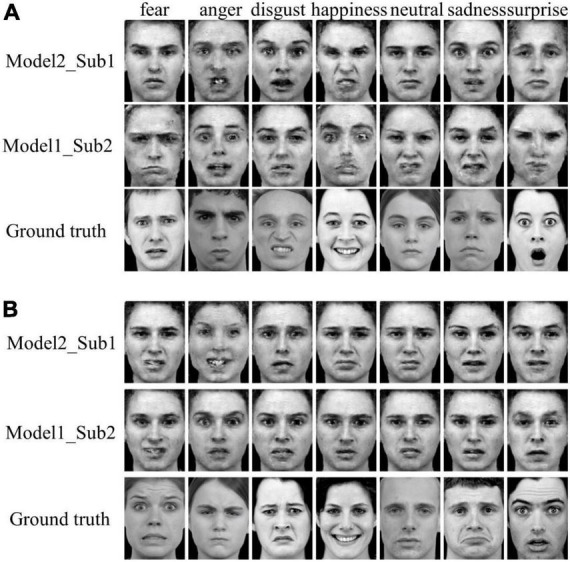
Inter-subject face reconstruction from functional magnetic resonance imaging (fMRI) signals. **(A)** Reconstruction of seen faces from brain activities in both subjects. **(B)** Reconstruction of unseen faces from brain activities in both subjects. Model2_Sub1: Reconstruction of faces from subject 1’s brain activities using the framework trained with subject 2’s fMRI data **(top row)**; Model1_Sub2: Reconstruction of faces from subject 2’s brain activities using the framework trained with subject 1’s fMRI data **(middle row)**. Ground truth: original face image stimuli **(bottom row)**.

We first used the fMRI responses to the 140 face stimuli from subject 1’s 5 run data to train the framework and the fMRI responses to the same face stimuli from subject 2’s data to reconstruct the perceived faces. The process was then exchanged between the two subjects, i.e., the fMRI response from subject 2 was used to train the framework and the fMRI response from subject 1 was used for reconstruction. By visualizing the reconstructed faces (Figure 6A), we found that although the face image quality is not ideal, all faces present recognizable expression, identity, and gender. The results from the evaluation metrics (see SSIM evaluation in Table 7A and all three evaluation metrics results of SSIM, MSE, and PSNR in Supplementary Table 5) showed that face images can be significantly reconstructed from subject 1’s fMRI data using the framework trained with subject 2’s fMRI signal, however, the reverse is not true (one-sample *t*-test, *p* = 0.032 for subject1, *p* = 0.91 for subject2, FDR corrected). According to the empirical scores, we found none of the three facial attributes can be significantly recognized (one-sample *t*-test, subject1: expression *p* > 0.99, identity *p* > 0.99, gender *p* = 0.96; subject2: expression *p* > 0.99, identity *p* > 0.99, gender *p* = 0.46, FDR corrected).

**TABLE 7:** Quantitative evaluation of seen (A) and unseen (B) face image reconstruction from inter-subject functional magnetic resonance imaging (fMRI) signals.

		Objective evaluation	Subjective evaluation
	
		SSIM	empirical scores (exp, id, and gen)
A	Model2_Sub1	0.35 ± 0.08	(3.32, 3.09, 4.44)
	Model1_Sub2	0.36 ± 0.08	(2.88, 2.81, 5.03)
B	Model2_Sub1	0.37 ± 0.07	(3.44, 2.85, 4.36)
	Model1_Sub2	0.38 ± 0.07	(3.63, 2.65, 4.39)

Model2_Sub1 means training the framework with the fMRI response of subject 2 and predicting the faces with subject 1. Model1_Sub2 means training the framework with the fMRI response of subject 1 and predicting the face images with subject 2.

exp, expression; id, identity; gen, gender.

*p < 0.05.

##### Reconstruction of unseen images

We also examined the inter-subject unseen face reconstruction (see Figure 6B). The fMRI responses to a subset of face stimuli from one of the subjects were used to train the framework and the fMRI responses to the remaining face stimuli from another subject were used for face reconstruction. We found no significant reconstruction performance either quantitative assessment or subjective evaluation of empirical scores (see SSIM evaluation in Table 7B and all three evaluation metrics results of SSIM, MSE, and PSNR in Supplementary Table 6).

Taken together, these results indicated that the proposed framework can only reconstruct faces across subjects to a certain extent, and there is still room for our proposed reconstruction framework to improve, when facing fMRI data from different stimulus sources and different subjects.

#### Contributions of brain regions

Though our current study addresses the methodology of face reconstruction from brain signals, the neural responses of brain signals at different local brain regions are decisive factors determining the reconstruction performance. As a typical kind of brain decoding method, perceived faces reconstruction provides an effective way to vividly explore the neural substrates of face perception. Here, we used our framework to estimate the contribution of each brain ROI to the reconstructed faces representing multiple attributes of facial expression, identity, and gender, to further explore the neural mechanism of multiple facial attribute perception in different regions of human visual cortex.

Here, we identified a reconstruction accuracy. The fMRI responses from each brain ROI were extracted to predict the multi-dimensional face features of expression, identity and gender, respectively, using LR module. The predicted face features were then compared with the “ground truth” face features elicited from MTDLN. For each test image’s each facial attribute, we used Euclidean distance to measure the similarity between pairs of face features. Besides the comparison of its predicted face features with the ground truth, we also randomly chose a distractor image other than the test image, and compared the predicted face features with this distractor image’s face features with Euclidean distance. If the value of Euclidean distance between the predicted features and ground truth is lower than that of the value between the predicted features and distractor image’s features, we defined this face image’s facial attribute as recognizable, otherwise unrecognizable. The reconstruction accuracy was calculated as the proportion of successfully recognized facial attributes among all the 140 test images. The procedure was repeated 40 times and 40 reconstruction accuracy values were obtained for the statistical analysis.

**FIGURE 7:**
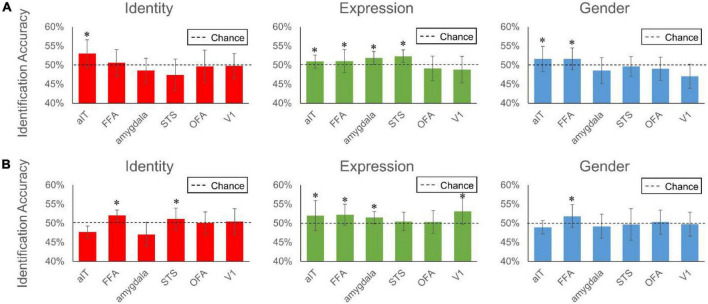
Reconstruction accuracy of each brain region of interest to facial expression, identity and gender. The two subplots represent performance from brain signals of Subject 1 **(A)** and Subject 2 **(B)**, respectively. Bars represent group-average accuracy (±SEM across 40 pairs). Black dashed line indicates the chance level reconstruction accuracy of 50%. *Indicates group-level significance *p* < 0.05.

We used the reconstruction accuracy of each ROI to represent the contribution of the ROI to the final reconstruction performance. Figure 7 shows the contribution of each of the six brain regions to the reconstruction of three facial attributes. As can be seen in the figure, FFA and aIT provided significant contributions to facial identity reconstruction (one-sample *t*-test, aIT: 53.00% for subject 1 with *p* < 0.001, FFA:52.04% for subject 2 with *p* < 0.001, FDR corrected), and pSTS and amygdala provided the significant contribution to facial expression reconstruction (one-sample *t*-test, pSTS: 52.29% for subject 1 with *p* < 0.001, amygdala: 51.85% for subject 1 with *p* < 0.001, amygdala: 51.45% for subject 2 with *p* < 0.001, FDR corrected). This result is consistent with many previous studies indicating that FFA and aIT were involved in facial identity encoding, and amygdala and pSTS were involved in encoding facial expression (Zhang et al., 2016). Meanwhile, the brain regions that showed significant contributions to facial identity reconstruction also showed significant contribution to gender reconstruction, which is not surprising, for gender can be understood as a special label of facial identity.

## Discussion

In our current study, we proposed a novel framework for reconstruction of perceived face images from brain activity. Our framework consists of three modules: (i) an optimized multi-task deep learning network, which is used to simultaneously extract the multi-dimensional face features attributed to facial expression, identity and gender from one single face image, (ii) a set of linear regression models, which are used to establish the mappings between the brain signals and the multi-dimensional face features, (iii) a multi-conditional generative adversarial network, which is used to generate the face images constrained by the predicted multi-dimensional face features. The experimental results demonstrated that the proposed framework can achieve the state-of-the-art reconstruction performance of both seen and unseen face images, and the reconstruction performance of face images was greatly improved than that of the traditional methods in both visual effects and quantitative assessment.

One contribution in our current study is that we optimized a multi-task deep neural network model, which we termed as “MTDLN”, to extract the multi-dimensional face features from one single face image. In VanRullen and Reddy (2019), VAE model was used to extract the face features from face image dataset, the face features were finally entered into a conditional GAN model for generating perceived faces. However, there is still a gap between their reconstructed faces and the ground truth, especially in representing certain kinds of facial attributes such as expression and gender. In this respect, our work goes beyond VanRullen et al. in exacting the facial attributes. Compared to four commonly used feature extraction methods, our experiment results demonstrated that our MTDLN module achieved substantial improvements in face feature extraction. Moreover, our method could clearly reconstruct perceived face images from the brain signals, especially in representing multiple facial attributes: expression, gender, and identity.

Another contribution is that our framework builds a bridge between different brain regions in human visual cortex and multi-dimensional face features representing multiple facial attributes contained in the face images. Existing methods often ignore the multiple facial attributes represented in face images, which evoke very different neural response patterns in different brain regions of the visual cortex. We thus established a set of linear relationships between brain signals of these ROIs and the multi-dimensional face features representing these facial attributes to characterize these mappings. The results showed that the selective use of brain signals from attribute-sensitive brain regions improved the face reconstruction. In addition, we also explored the contribution of different brain ROIs to the perceived face reconstruction. Our result indicated that pSTS and amygdala provided significant contribution to facial expression reconstruction, and FFA and aIT provided significant contribution to facial identity and gender reconstruction. These findings provide strong evidence supporting the neural theory that facial attributes of expression and identity are processed in two distinct neuroanatomical pathways in primate visual cortex (Zhang et al., 2016), thus helping us better understand the neural mechanism underlying the face perception.

Besides, we also used our framework to estimate the contribution of each brain ROI to the reconstructed faces representing multiple attributes of facial expression, identity, and gender. Our results showed that FFA and aIT provided significant contributions to facial identity reconstruction, and pSTS and amygdala provided the significant contribution to facial expression reconstruction. Many previous studies have also explored the coding of face-selective areas to different facial attributes. Pessoa et al., 2002, 2006 performed fMRI studies in healthy subjects and found that amygdala was able to accurately discriminate between different expressions. Using transcranial magnetic stimulation (TMS), Pitcher (2014) found that the pSTS and rOFA contributes to facial expression recognition. Zhang et al. (2016) used fMRI and support vector machine pattern classification analysis to reveal that the decoding of facial emotion and facial identity occurs in different neural substrates: the amygdala and STS for the former and FFA and aIT for the latter. Shao et al. (2017) used condition-rich and single-image analysis approach to conclude that FFA showed high face-category selectivity, pSTS showed evidence of face-exemplar sensitivity, and OFA might be a transitional stage between general and face-selective information processing. Our result is consistent with these studies indicating that FFA and aIT were involved in coding facial identity, and amygdala and pSTS were involved in coding facial expression (Pessoa et al., 2002, 2006; Pitcher, 2014; Zhang et al., 2016; Shao et al., 2017). Meanwhile, the brain regions that showed significant contributions to facial identity reconstruction also showed significant contribution to gender reconstruction, which is not surprising, for gender can be understood as a special label of facial identity. Thus, our results provide strong evidence for the idea of dissociation of neural pathways mediating facial expression and identity discrimination in the human brain (Bruce and Young, 1986; Young et al., 1993; Haxby et al., 2000).

Our framework cannot only reconstruct the seen face images (the testing face images appear in training the framework parameters while their corresponding fMRI signals are from different scan runs), but also achieve excellent reconstruction performance for the unseen face images (the testing face images do not appear in training our framework parameters). This is due to the fact that we constructed a multi-dimensional face feature space based on a large sample size of multi-label face dataset, representing multiple facial attributes of expression, identity, gender, and theoretically, any single face image’s three attributes could be well represented in the face space, and therefore an excellent face reconstruction effect could be achieved based on the face features predicted by the fMRI signal even if they are unseen face images. In addition, although our method achieved outstanding performance on intra-subjects, it does not work very well on inter-subjects perceived face reconstruction, which may be due to two factors: first, the neural response patterns elicited by the same face images on different subjects are too different, and the current method we use is not sufficient to achieve such inter-subject face reconstruction; second, the quality of the fMRI signal differs among different subjects. Therefore, how to achieve inter-subject face reconstruction and make our reconstruction framework more universal and practically applicable is a key point of our next research.

## Data availability statement

Publicly available datasets were analyzed in this study. This data can be found here: The fMRI data for this study is available from the authors upon request. Codes can be obtained from https://github.com/round99/Perceived-face-reconstruction.

## Ethics statement

The studies involving human participants were reviewed and approved by the Research Ethics Committee at Institute of Biophysics, Chinese Academy of Sciences. The patients/participants provided their written informed consent to participate in this study. Written informed consent was obtained from the individual(s) for the publication of any potentially identifiable images or data included in this article.

## Author contributions

XH: methodology, software, and writing and evaluation. JZ: software, evaluation, and writing—review and editing. HZ: conceptualization, supervision, and writing—review and editing. All authors contributed to the article and approved the submitted version.
