# Supplementary material for: Reconstruction of perceived face images from brain activities based on multi-attribute constraints
Source: Front Neurosci. 2022 Oct 26;16:1015752. doi: 10.3389/fnins.2022.1015752 (PMC9643433; doi:10.3389/fnins.2022.1015752)
Supplement: Supplementary file 1 [file Data_Sheet_1.pdf]

Supplementary Material

1 Supplementary Tables

Table S1. Quantitative evaluation (MSE, SSIM and PSNR) of reconstruction performance by using 5 different face-feature extraction models. exp: expression, id: identity, gen: gender. * means p<0.05.

Methods	Objective Evaluation			Subjective Evaluation
	MSE	SSIM	PSNR	Empirical scores (exp, id, gen)
PCA	2958.98±828.25	0.37±0.05*	13.58±1.18	(2.69, 2.65, 3.44)
VAE	2687.24±2032.36	0.51±0.08*	14.90±3.01	(4.95, 4.40, 6.00*)
pre-trained VGG-Face	2834.90±791.55	0.45±0.06*	13.78±1.27	(6.25*, 5.58, 7.46*)
re-trained VGG-Face	4570.90±1621.20	0.21±0.06	11.80±1.55	(1.22, 1.01, 1.31)
MTDLN	2050.66±760.42	0.53±0.06*	15.28±1.53	(8.50*, 8.10*, 9.17*)

Table S2. Quantitative evaluation (MSE, SSIM and PSNR) of reconstruction performance with three different ROI combination strategies. exp means expression, id means identity, gen means gender. * means p<0.05.

	Brain ROIs	Subjects	MSE	SSIM	PSNR
Strategy1	id/gen/exp: V1	Sub1	4034.26±1473.18	0.36±0.08	12.33±1.49
		Sub2	3867.16±1304.54	0.36±0.07	12.48±1.37
Strategy2	id/gen/exp: V1, OFA, amygdala, STS, FFA, aIT	Sub1	3825.59±1490.96	0.38±0.08	12.59±1.54
		Sub2	3751.19±1426.67	0.37±0.07	12.65±1.47
Our proposed strategy	id/gen: V1, OFA, FFA, aIT exp: V1, OFA, amygdala, STS	Sub1	3494.53±1451.89	0.39±0.08*	13.01±1.61
		Sub2	3479.34±1348.08	0.38±0.07*	12.97±1.41

Table S3. Quantitative reconstruction evaluation of seen face images from intra-subject fMRI signals. exp means expression, id means identity, gen means gender. * means p<0.05.

	Objective Evaluation			Subjective Evaluation
	MSE	SSIM	PSNR	Empirical scores (exp, id, gen)
Sub1	3494.53±1451.89	0.39±0.08*	13.01±1.61	(5.27, 3.74, 6.10*)
Sub2	3479.34±1348.08	0.38±0.07*	12.97±1.41	(3.89, 4.05, 6.43*)

Table S4. Quantitative reconstruction evaluation of unseen face images from intra-subject fMRI signals. exp means expression, id means identity, gen means gender. * means p<0.05.

	Objective Evaluation			Subjective Evaluation
	MSE	SSIM	PSNR	Empirical scores (exp, id, gen)
Sub1	3182.66±1095.39	0.40±0.06*	13.34±1.41	(4.83, 2.96, 4.38)
Sub2	3306.02±1132.21	0.38±0.07*	13.17±1.41	(3.96, 4.00, 6.38*)

Table S5. Quantitative reconstruction evaluation of seen face images from inter-subject fMRI signals. Model2_Sub1 means training the framework with the fMRI response of subject 2 and predicting with subject 1. Model1_Sub2 means training the framework with the fMRI response of subject 1 and predicting with subject 2. exp means expression, id means identity, gen means gender. * means p<0.05.

	Objective Evaluation			Subjective Evaluation
	MSE	SSIM	PSNR	Empirical scores (exp, id, gen)
Model2_Sub1	3555.15±1275.24	0.35±0.08	12.87±1.45	(3.32, 3.09, 4.44)
Model1_Sub2	3544.78±1231.33	0.36±0.08	12.87±1.39	(2.88, 2.81, 5.03)

Table S6. Quantitative reconstruction evaluation of unseen face images from inter-subject fMRI signals. Model2_Sub1 means training the framework with the fMRI response of subject 2 and predicting with subject 1. Model1_Sub2 means training the framework with the fMRI response of subject 1 and predicting with subject 2. exp means expression, id means identity, gen means gender. * means p<0.05.

	Objective Evaluation			Subjective Evaluation
	MSE	SSIM	PSNR	Empirical scores (exp, id, gen)
Model2_Sub1	3526.12±1182.93	0.37±0.07	12.89±1.40	(3.44, 2.85, 4.36)
Model1_Sub2	3502.03±1199.12	0.38±0.07	12.92±1.40	(3.63, 2.65, 4.39)

Table S7. Quantitative evaluation of seen face images from intra-subject fMRI signals by using 5

different methods. exp: expression, id: identity, gen: gender. * means p<0.05.

Methods		Objective Evaluation SSIM	Subjective Evaluation Empirical scores (exp, id, gen)
PCA	Sub1	0.31±0.06	(2.57,0.70,3.47)
	Sub2	0.31±0.06	(2.53,0.63,3.43)
VAE	Sub1	0.37±0.09*	（4.13,3.63,6.00*）
	Sub2	0.34±0.09*	(2.95, 3.33, 5.57*)
pre-trained VGG-Face	Sub1	0.37±0.08*	（4.10,3.59,5.85*）
	Sub2	0.33±0.07*	(2.88, 3.04, 5.18)
re-trained VGG-Face	Sub1	0.18±0.07	(0.62,0.37,0.77)
	Sub2	0.18±0.07	(0.64,0.36,0.69)
MTDLN	Sub1	0.39±0.08*	(5.27,3.74,6.10*)
	Sub2	0.38±0.07*	(3.89, 4.05, 6.43*)

Table S8. Quantitative evaluation of unseen face images from intra-subject fMRI signals by using 5 different methods. exp: expression, id: identity, gen: gender. * means p<0.05.

Methods		Objective Evaluation SSIM	Subjective Evaluation Empirical scores (exp, id, gen)
PCA	Sub1	0.30±0.06	(2.20,0.60,3.13)
	Sub2	0.30±0.06	(2.17,0.59,3.06)
VAE	Sub1	0.37±0.04*	（4.39,2.63,4.10）
	Sub2	0.35±0.04*	(3.83,2.30,4.21)
pre-trained VGG-Face	Sub1	0.38±0.07*	（4.37,2.58,4.05）
	Sub2	0.36±0.07*	(3.93,2.37,4.34)
re-trained VGG-Face	Sub1	0.18±0.06	(0.53,0.33,0.65)
	Sub2	0.18±0.06	(0.52,0.34,0.68)
MTDLN	Sub1	0.40±0.06*	(4.83,2.96,4.38)
	Sub2	0.38±0.07*	(3.96,4.00,6.38*)

Table S9. Quantitative evaluation of reconstructed face images predicted from noise and face feature. exp: expression, id: identity, gen: gender. * means p<0.05.

Input	Objective Evaluation SSIM	Subjective Evaluation Empirical scores (exp, id, gen)
Noise	0.26±0.07	(1.57,0.43,2.71)
Feature	0.53±0.06*	(8.50*,8.10*,9.17*)

Table S10. Talairach coordinates of face-selective regions for Subject 1 (A) and Subject 2 (B).

	ROI		x	y	z
A	FFA	Left	-44	-55	-17
		Right	47	-56	-22
	OFA	Left	-41	-79	-15
		Right	39	-78	-12
	aIT	Left	-29	-4	-37
		Right	31	-4	-36
	pSTS	Left	-50	-53	13
		Right	42	-55	13
	Amygdala	Left	-24	-7	-12
		Right	24	-7	-13
B	FFA	Left	-35	-45	-11
		Right	44	-41	-22
	OFA	Left	-38	-72	-19
		Right	41	-69	-13
	aIT	Left	-38	-4	-40
		Right	36	-2	-44
	pSTS	Left	-48	-63	11
		Right	48	-56	7
	Amygdala	Left	-17	-3	-14
		Right	22	-3	-14

Table S11. Quantitative evaluation of reconstruction performance with five different attributes constraints strategies. exp means expression, id means identity, gen means gender. * means p<0.05.

	Brain ROIs	Constraints	Subjects	MSE	SSIM	PSNR
Strategy 3	id/gen/exp: V1, OFA,amygdala, STS, FFA, aIT	id	Sub1	5983.09± 1482.85	0.08±0.03	10.48±1.01
			Sub2	5930.16± 1519.74	0.09±0.03	10.53±1.05
Strategy 4	id/gen/exp: V1, OFA,amygdala, STS, FFA, aIT	id&gen	Sub1	3859.4±1 526.18	0.35±0.07	12.56±1.55
			Sub2	3889.17± 1509.62	0.34±0.07	12.53±1.59

Strategy 5	id/gen/exp: V1, OFA,amygdala, STS, FFA, aIT	id&emo	Sub1	3459.0±1 591.48	0.37±0.08	13.12±1.74
			Sub2	3450.96± 1403.35	0.36±0.07	13.05±1.58
Strategy 2	id/gen/exp: V1, OFA,amygdala, STS, FFA, aIT	id&gen&emo	Sub1	3825.59± 1490.96	0.38±0.08	12.59±1.54
			Sub2	3751.19± 1426.67	0.37±0.07	12.65±1.47
Our proposed strategy	id/gen: V1, OFA, FFA, aIT exp: V1, OFA, amygdala, STS	id&gen&emo	Sub1	3494.53± 1451.89	0.39±0.08*	13.01±1.61
			Sub2	3479.34± 1348.08	0.38±0.07*	12.97±1.41

2 Supplementary Figures

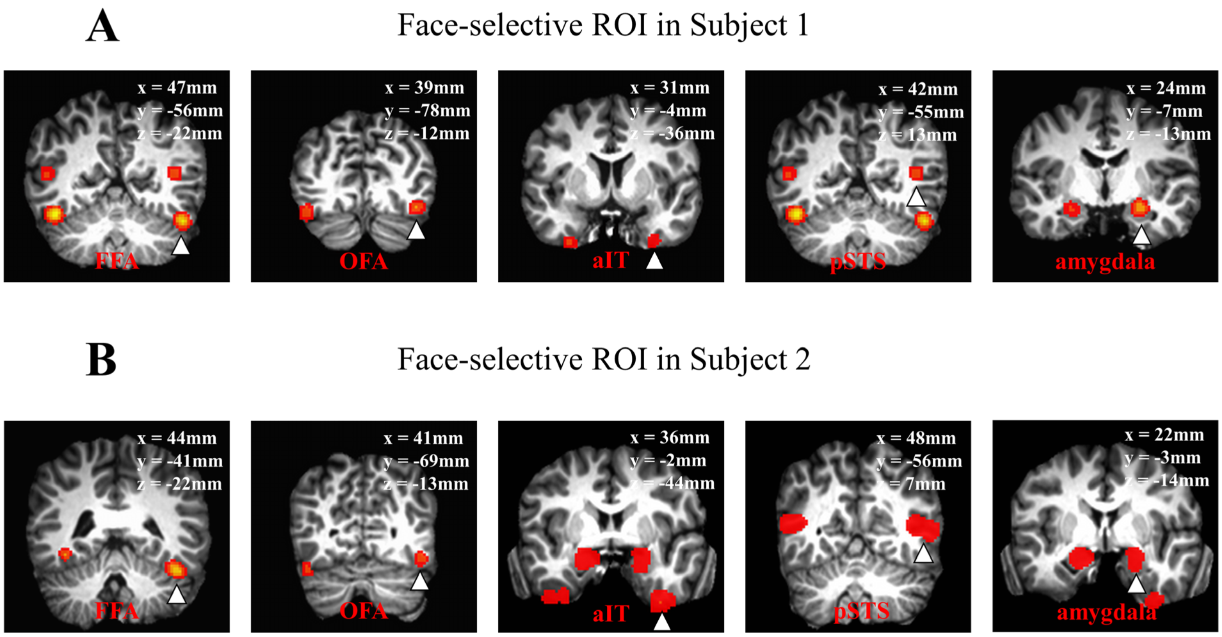

Figure S1. Face-selective ROI in Subject 1 and Subject 2.

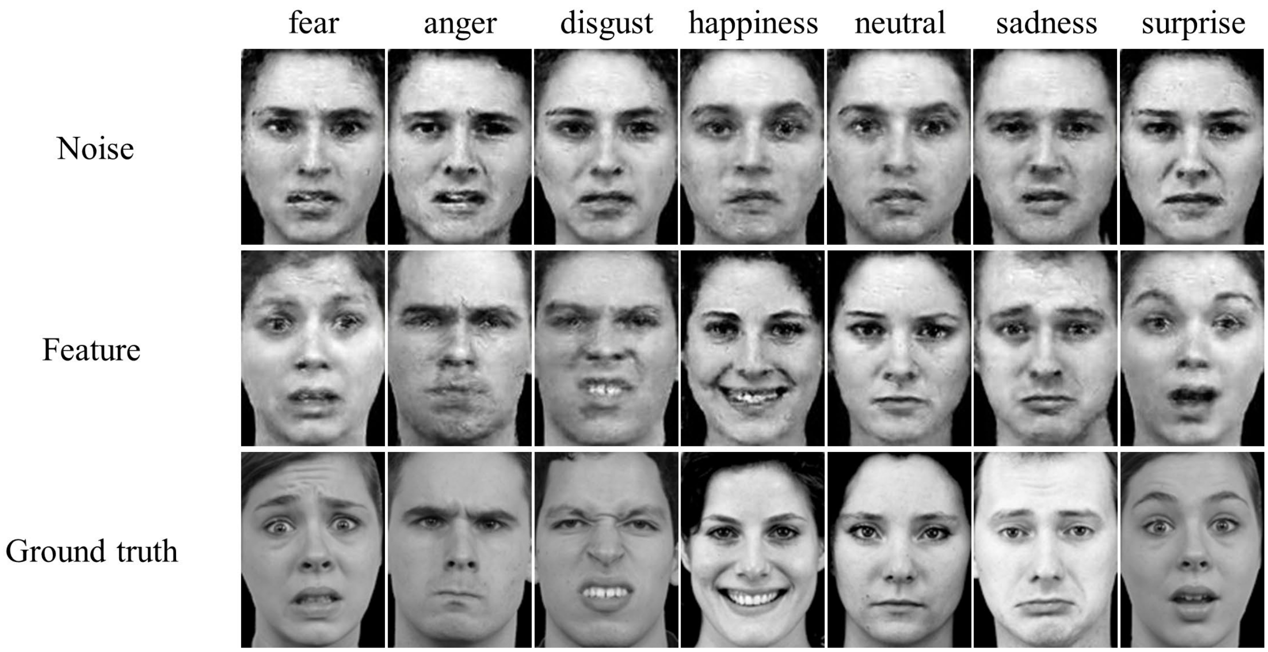

Figure S2. Representative samples of reconstructed faces predicted from noise and feature. From top row to bottom row: Noise, Feature and Ground truth.

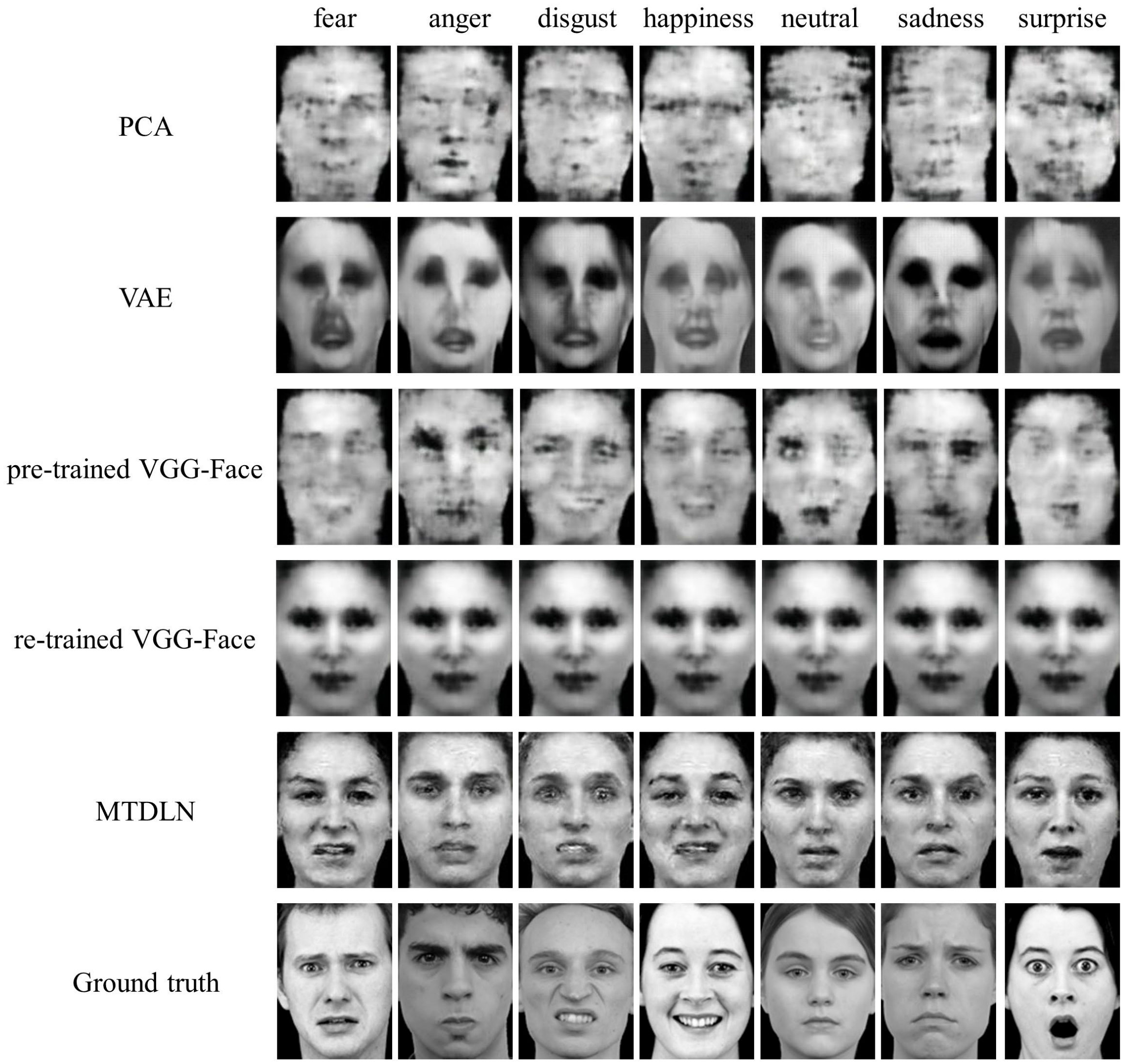

Figure S3. Representative samples of reconstructed seen faces from fMRI signals of subject 1 with 5 different methods. From top row to bottom row: PCA, VAE [19], pre-trained VGG-Face, re-trained VGG-Face, MTDLN and Ground truth.

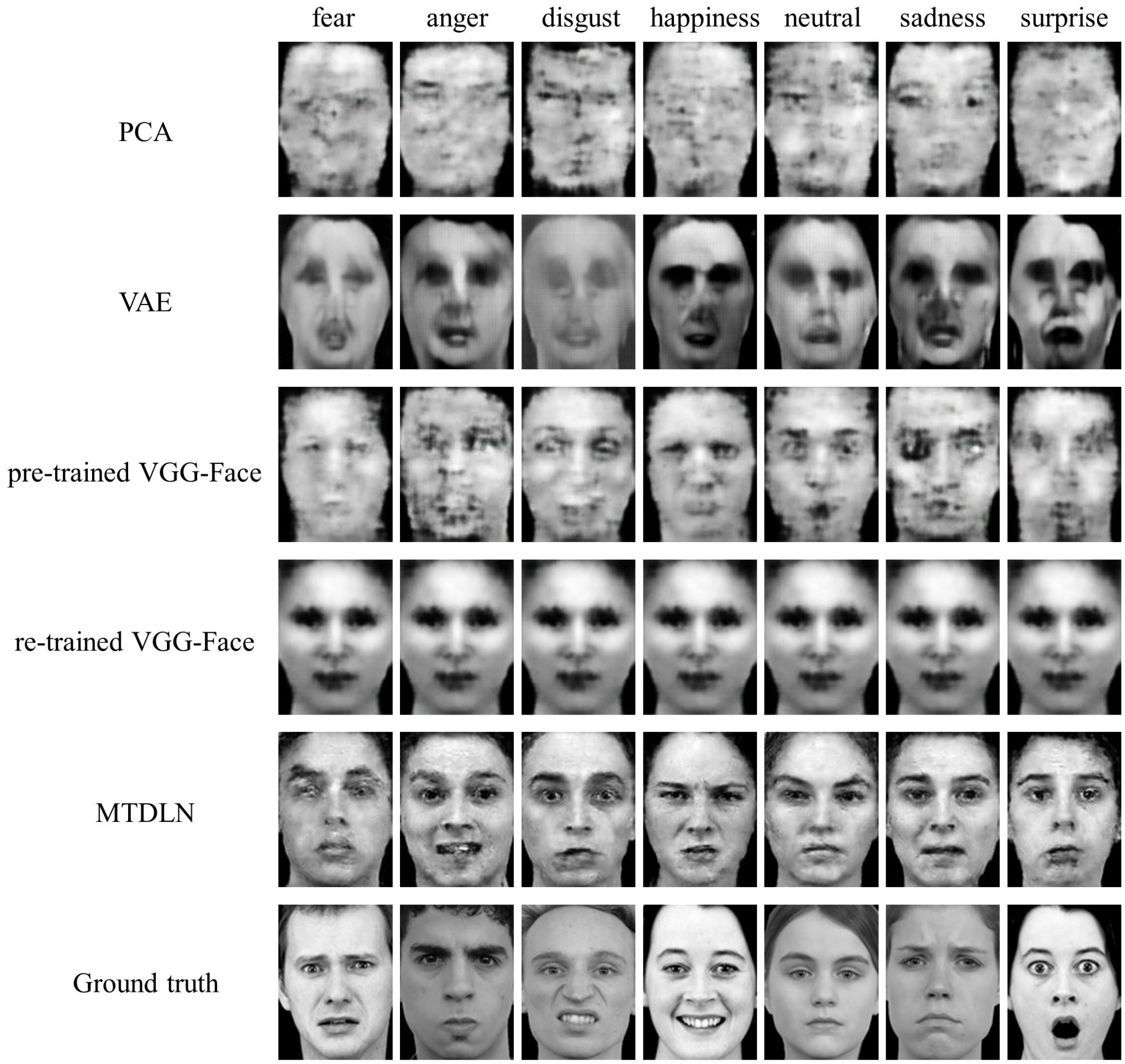

Figure S4. Representative samples of reconstructed seen faces from fMRI signals of subject 2 with 5 different methods. From top row to bottom row: PCA, VAE [19], pre-trained VGG-Face, re-trained VGG-Face, MTDLN and Ground truth.

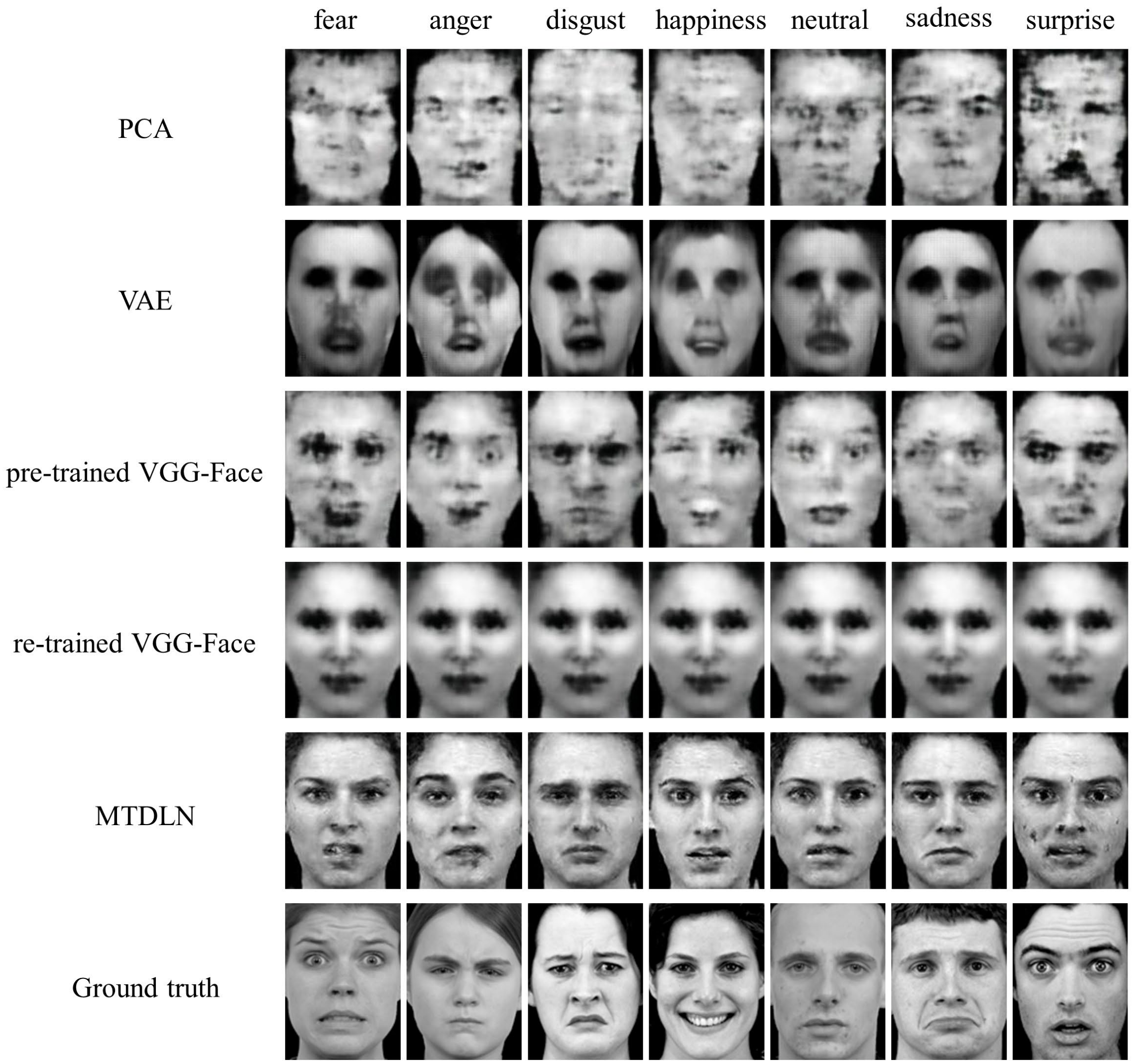

Figure S5. Representative samples of reconstructed unseen faces from fMRI signals of subject 1 with 5 different methods. From top row to bottom row: PCA, VAE [19], pre-trained VGG-Face, re-trained VGG-Face, MTDLN and Ground truth.

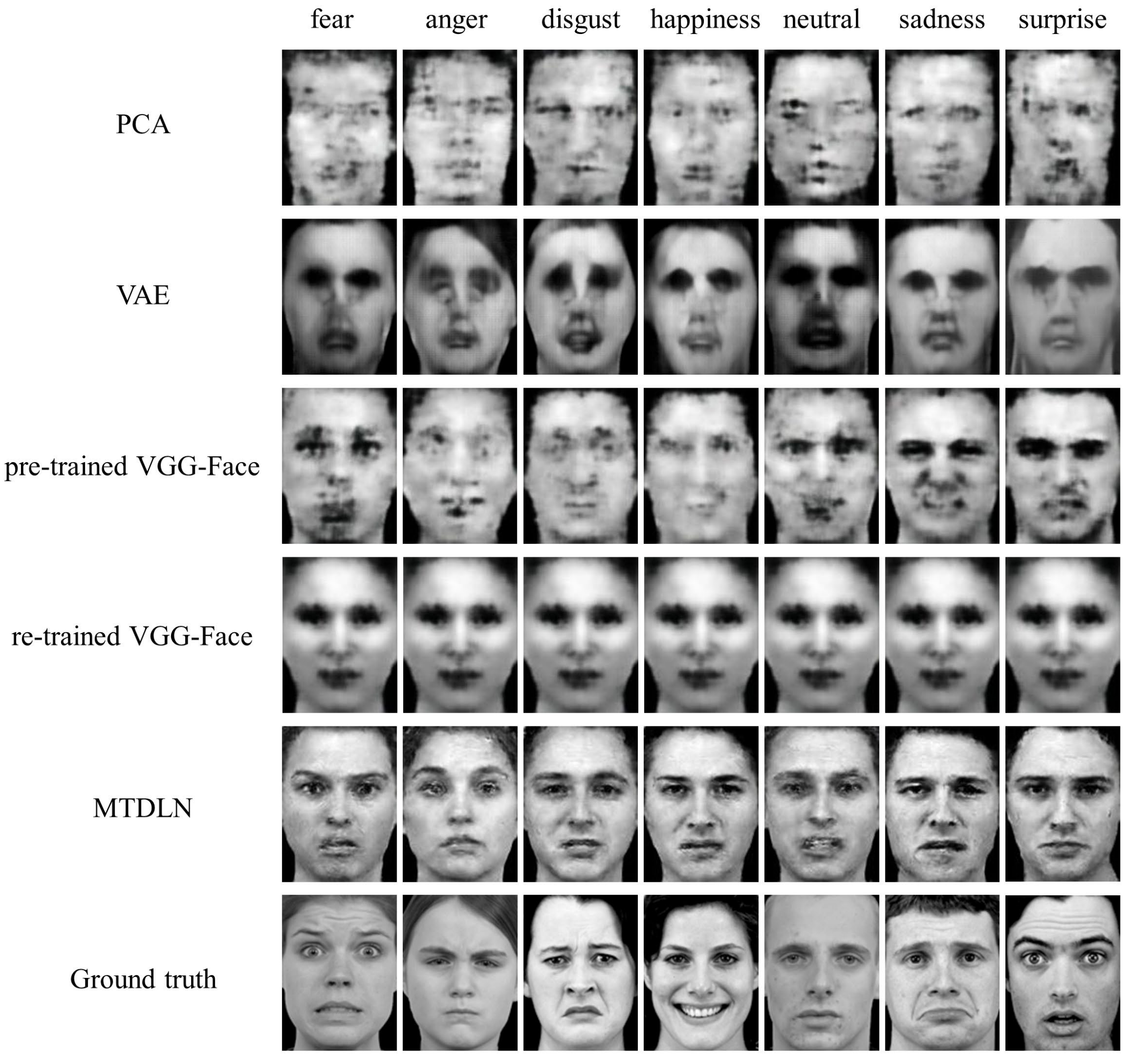

Figure S6. Representative samples of reconstructed unseen faces from fMRI signals of subject 2 with 5 different methods. From top row to bottom row: PCA, VAE [19], pre-trained VGG-Face, re-trained VGG-Face, MTDLN and Ground truth.
